# Dry needling for the treatment of acute myofascial pain syndrome in general practitioners’ clinics: a cohort study

**DOI:** 10.1186/s12875-022-01951-0

**Published:** 2022-12-27

**Authors:** Ilan Yehoshua, Oded Rimon, Miri Mizrahi Reuveni, Roni Peleg, Limor Adler

**Affiliations:** 1 Department of Family Medicine, Maccabi Healthcare Service, 27 Hamered Street, Tel Aviv, Israel; 2 Ben Gurion University, Beer Sheva, Israel; 3 Sackler Faculty of Medicine, Tel Aviv University, Tel Aviv, Israel

**Keywords:** Dry needling, Myofascial pain syndrome, Primary health care, General practice

## Abstract

**Background:**

Musculoskeletal pain is one of the leading complaints in the ambulatory setting. There are many ways to treat it, including pharmacologic and non-pharmacologic approaches. Dry needling (DN) is an option that is easy to learn, cheap and has a good safety profile. The aim of this study was to assess the association between DN performed by GPs for acute myofascial pain syndrome (MPS) and pain relief and to evaluate factors associated with treatment success.

**Methods:**

In this prospective cohort study, two GPs performed DN in their clinics. Patients were asked to rank their pain using the Short-Form McGill Pain Questionnaire (SF-MPQ) before, 10-min and 1-week after the procedure. The SF-MPQ index consists of 3 parts; visual analog scale (VAS), pain rating index (PRI) and present pain intensity (PPI).

Logistic regressions were performed to assess the variables associated with short- and medium- term success.

**Results:**

Fifty two patients were recruited from September 2019 until August 2020. VAS was 6.0 ± 2.3 (before), 4.1 ± 2.5 (10-min after) and 2.6 ± 2.71 (1-week after), *P* < 0.05. PRI was 17 ± 9.1 (before), 10.8 ± 8.5 (10-min after) and 5.1 ± 6.5 (1-week after), *P* < 0.05. PPI was 2.6 ± 1.0 (before), 1.7 ± 1.0 (10-min after) and 1.1 ± 1.2 (1-week after), P < 0.05.

Short-term success was associated with the physician who performed the procedure (OR 10.08, 95% CI 1.15,88.4) and with the use of a single needle (vs. multiple needles inserted) (OR 4.55, 95% CI 1.03,20.11). Medium-term success was associated with being a native born (non-immigrant), OR 8.59, 95% CI 1.11,66.28 and with high level of initial pain, OR 11.22, 95% CI 1.82,69.27.

**Conclusion:**

Our study demonstrated improvement in acute pain 10-min and 1-week after DN performed by a GP, in all parts of the SF-MPQ. Therefore, we believe DN is a good therapeutic option for GPs to aid patients suffering from MPS.

**Supplementary Information:**

The online version contains supplementary material available at 10.1186/s12875-022-01951-0.

## Background

Pain is one of the leading complaints in the ambulatory setting. The Global Burden of Diseases, Injuries, and Risk Factors Study 2019 demonstrated that low back pain, neck pain and other musculoskeletal pain complaints are the leading causes of morbidity globally in terms of years lived with disease [1]. Musculoskeletal pain is one of the most frequent complaints in general practitioners’ (GPs) clinics [2].

Myofascial pain syndrome (MPS) is a potential cause of musculoskeletal pain [3]. MPS is defined as a regional pain characterized by the presence of myofascial trigger points (MTrPs) [4, 5, 3]. MtrPs is a hyperirritable palpable nodule in the skeletal muscle fibers that can produce local or referred pain. There are many ways to treat MPS, including pharmacologic and non-pharmacologic approaches [6]. Dry needling (DN) is an option that is easy to learn, cheap and has a good safety profile [7]. DN is performed by inserting a needle into MtrPs located in skeletal muscles. It is a simple method to deal with pain in the physician’s office.

The effectiveness of DN was examined in many studies. Most systemic reviews and meta-analysis recommended DN for treating acute and chronic pain in patients with temporomandibular joint dysfunction [8], upper-quarter myofascial pain [9], upper trapezius pain [10], neck and shoulder pain [11–15], elbow pain [16], low back pain [17–19], knee pain [20], and plantar fasciitis [21]. On the other hand, some studies have shown no efficacy for DN treatment in MPS [22], specifically in the neck [23] and upper extremities [24].

Side effects of dry needling include minor adverse events such as mild bleeding (16%), bruising (7.7%) and pain during DN (5.9%). Major adverse events such as pneumothorax or hemothorax are rare (< 0.1%) [7].

In 2015, the Israeli society of Musculoskeletal Medicine released a position paper concerning intra-muscular stimulation (IMS), written by six experts, using the Delphi procedure [25]. The general statement agreed upon was “IMS is one of the preferred treatments for MPS. The treatment is evidence-based, effective, safe, and inexpensive. The position of the Israeli Society of Musculoskeletal Medicine is that the treatment should be taught and used by all GPs and those physicians in other areas of medicine who deal with pain in their work.”

In 2017, Maccabi Healthcare Services, the second largest health maintenance organization (HMO) in Israel, launched DN courses for GPs. The basic course consists of 3 sessions of 10 academic hours each. Every GP who attends the course is afterwards certified to treat their patients with DN. Since, there has been an increase in the number of certified physicians, from 107 in 2017 to 199 in 2020 as well as an additional increase in the number of DN procedures, from 7,644 in 2017 to 10,647 in 2020 (a slight decrease compared to 2019, during which 12,088 DN procedures were done; this decrease might be due to the COVID-19 effect).

The aim of this study was to assess the association between DN performed by GPs for acute MPS and short- and medium-term pain relief. Secondary aim was to evaluate factors that are associated with treatment success.

## Methods

### Study design

This is a prospective cohort study. Two GPs who regularly treat patients with DN in their clinics invited eligible patients to participate in the study. Participation was voluntary and patients who refused to take part were still treated with DN. Patients who agreed to take part in the study were given full explanation on the procedure of DN and possible side effects and signed a written informed consent statement. Patients were asked to rank their pain using the Short-Form McGill Pain Questionnaire (SF-MPQ) before, 10 min after and 1 week after the procedure. The study was approved by the ethical committee of Bait Balev (the institutional review board), ID 0017–19-BBL. All data was saved anonymously.

### Setting

DN was performed by two GPs, one is a specialist with 3 years of experience with DN and 8 years working in the same clinic and the other a resident, with 4 years of experience with DN but only 1 year working in the clinic. The GPs who performed DN chose the muscles to be treated based on the patient's complaints and findings of the physical examination. The length of the needle used for DN varies according to the muscle location, from 30 to 120 mm, and the diameter was 0.3 mm. Insertion of the needle was deep and perpendicular except for the back area, where it was oblique to the skin.

### Study population

Inclusion criteria were age > 18 years old, Hebrew speakers, acute MPS (less than a week from onset) and consent to take part in the study. Exclusion criteria were pregnancy, local infection, hemophilia, anti-coagulants use, local mass in the site of insertion, a contagious disease (hepatitis B, hepatitis C, HIV), having orthopedic implants or having undergone lymph node dissection. Follow-up of patients was done via telephone by the lead researcher.

The diagnosis of MPS in this study relies on specific finding in the physical examination and clinical judgment of the physicians. The findings relevant for MPS in the physical examination include identification of a taut band and intentionally producing the pain by application of pressure to a point of tenderness within the band [26, 27].

### Variables

The outcome variables were the SF-MPQ which consists of three distinct parts; First, pain rating index – which includes 15 words describing pain (in 2 subscales – sensory and affective), each rated on intensity scale of 0 (none) to 3 (severe). Total scores range from 0 to 45. Second, present pain intensity which represents the amplitude of pain from 0 (no pain) to 5 (excruciating pain) and third, a 10 cm visual analog scale (VAS) for average pain [28, 29]. The SF-MPQ is considered a reliable tool to indicate clinically valuable difference in musculoskeletal pain [30].

Independent variables included were sociodemographic and DN related variables; sociodemographic data were collected for all participants including gender, age, marital status, country of birth (native born vs. other), socioeconomic status (SES, rated from 1 (lowest) to 10, (highest)). Smoking status and DN related variables were also collected, including exact site of DN, single vs. multiple needles inserted, the physician who performed the procedure and the baseline VAS (low   [1,2,3,4,5] and high [3,6,7,8,9]).

### Statistical analysis

Sample size was calculated based on assumptions of pain rating index of 30 before DN and 25 after DN (with SD of 7) and VAS of 6 before DN and 4 after DN with a power of 90%, p value < 0.001 and a correlation of 0.4 assumed between both tests. These assumptions yield a sample size of 50 patients.

For each part of the SF-MPQ a mean and standard deviation were produced (pain rating index, present pain intensity and VAS; before DN, 10-min and 1-week after DN). Change was estimated using ANOVA with Bonferroni correction;10 min after compared to the baseline (short-term effect), 1-week after compared to the baseline (medium-term effect) and 1-week after compared to 10 min after. A success was defined has having at least 2 measures that have improved (out of the three parts of the SF-MPQ). A continuous success was considered success in both short- and medium-term indices. A logistic regression was performed to assess what variables are associated with short-term, medium-term, and continuous success, using the Forward approach. Analyses were performed using SPSS Statistics, version 27.

## Results

From September 2019 to August 2020, 55 patients were recruited (all data are available in the supplementary file). Three patients were excluded due to loss of follow-up at 1 week. 52% of patients were women with a mean age of 37.8 ± 12.6 (Table 1). Most of the DN procedures were done on the Trapezius muscle (40%), Iliocostalis Lumborum (27.3%) and Latissimus Dorsi (12.7). In each procedure one or more needles were inserted (76.9% single needle vs. 23.1% multiple needles).

**Table 1:** Characteristics of the patients recruited to the study

**Sociodemographic characteristics**	
Age	
mean ± SD	37.0 ± 12.2
range	18–62
	**n (%)**
Gender	
female	27 (51.9)
male	25 (48.1)
Birth country	
Israel	45 (86.5)
Other	7 (13.5)
SES	
1–4 – low	8 (15.4)
5–7 – reference	35 (67.3)
8–10—high	9 (17.3)
Marital status	
Single	35 (67.3)
Married	14 (26.9)
divorced	3 (5.8)
Smoking status	
Smoker	12 (23.1)
Non-smoker	40 (76.9)
**Dry Needling Procedure characteristics**	
Physicians	
#1 (specialist)	18 (34.6)
#2 (resident)	34 (65.4)
Needles inserted	
Single	40 (76.9)
multiple	12 (23.1)
Initial VAS	
> 5	33 (63.5)
≤ 5	19 (36.5)

### Univariate analysis

**Fig. 1:**
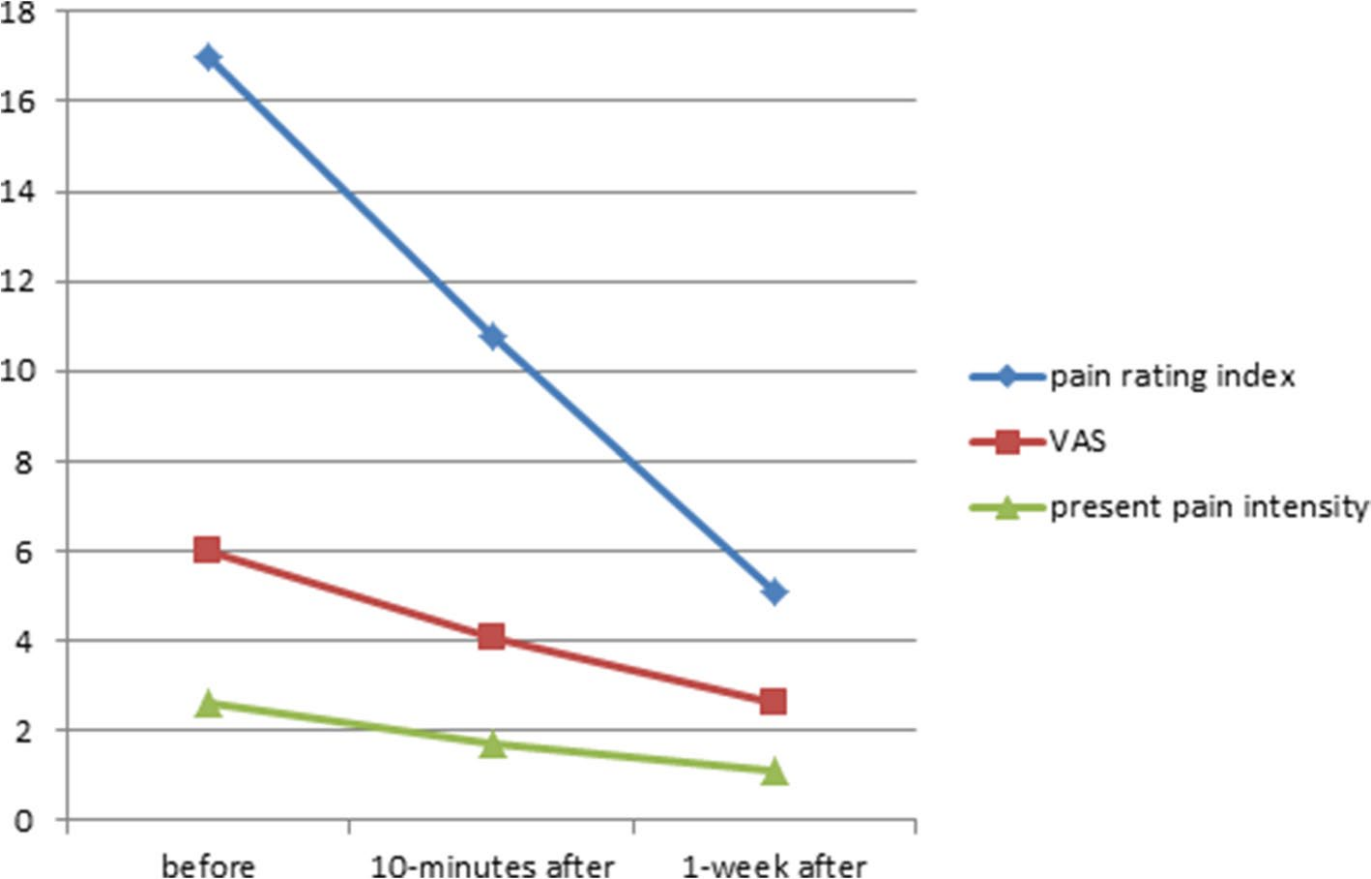
Change in all parts of the Short-Form McGill Pain Questionnaire

VAS was 6.0 ± 2.3 *before DN* was performed, 4.1 ± 2.5 *10-min after* and 2.6 ± 2.7*1-week after*. Pain rating index was 17 ± 9.1 *before DN* was performed, 10.8 ± 8.5 *10-min after* and 5.1 ± 6.5 *1-week after*. Present pain intensity was 2.6 ± 1.0 *before DN* was performed, 1.7 ± 1.0 *10-min after* (*P* < 0.001) and 1.1 ± 1.2 *1-week after* (Table 2, Fig. 1). All comparisons were significant with a *P* < 0.05.

**Table 2:** Univariate analysis of differences in pain indexes using ANOVA

	Before the intervention (a)	10-min after the intervention (b)	1-week after the intervention (c)	*P* value
Visual Analog Scale (VAS)	6.0 ± 2.3	4.1 ± 2.5	2.6 ± 2.71	(a),(b) < 0.001 (a),(c) < 0.001 (b),(c) 0.009
Pain Rating Index	17 ± 9.1	10.8 ± 8.5	5.1 ± 6.5	(a),(b) < 0.001 (a),(c) < 0.001 (b),(c) 0.001
Present Pain Intensity	2.6 ± 1.0	1.7 ± 1.0	1.1 ± 1.2	(a),(b) < 0.001 (a),(c) < 0.001 (b),(c) 0.020

### Multivariate analysis

A logistic regression for short-term success (improvement after 10-min) showed that 2 factors were associated with it; the physician who performed the procedure (OR 10.08, 95% CI 1.15,88.4) and single needle inserted (vs. multiple needles) (OR 4.55, 95% CI 1.03,20.11). Medium-term success (improvement after 1-week, compared to baseline) was associated with being a native born (i.e., not having immigrated to Israel) (OR 8.59, 95% CI 1.11,66.28) and high level of initial pain (OR 11.22, 95% CI 1.82,69.27). Continuous success was associated with insertion of one needle, compared to multiple needles (OR 5.00, 95% CI 0.97,25.77).

## Discussion

### Main results

Our study demonstrated improvement in acute MPS 10-min after and 1-week after DN. Improvement was demonstrated in all parts of the SF-MPQ (pain rating index, present pain intensity and VAS). Short-term success was associated with the physician who performed the procedure and the insertion of a single needle (vs. multiple needles). Medium-term success was associated with being a native born and with higher levels of baseline pain (as evident by a VAS level higher than 5). Continuous success was associated with insertion of one needle (vs. multiple needles).

### Interpretation

In this study we explored the effectiveness of DN when performed by GPs in their clinics. Most research conducted on DN focuses on other health care providers who perform this intervention (physiotherapists and consultant physicians). The only data we found about DN in family practice is a review published by the Journal of the American Board of Family Medicine in 2010 [31]. In this review, Kalichman & Vulfsons recommended that DN can be used as part of the complex treatment of musculoskeletal pain by GPs.

Our findings on the effectiveness of DN support the body of knowledge that DN can be considered as a good treatment for acute pain [32]. The success of DN in improving pain levels was reported immediately after and also had a delayed effect with better results reported 1-week later. This suggests that the effect is continuous. Previous studies have shown both short-term and medium-term positive effect on pain intensity when comparing DN to sham needling [33–35], and to no intervention [36]. We demonstrated a mean VAS decrease of 1.9 10-min after DN and 3.4 1-week after. These findings are in line with other studies on the effect of DN, which showed a mean VAS decrease larger than 1.5 [37]; this decrease is larger than 1.2, the minimum clinically important difference in VAS [38].

High intensity of pain before the procedure, as indicated by a VAS of 6 or more, was associated with medium-term success. This might suggest that DN is more effective in alleviating pain when it is severe in the first place.

We believe that certain aspects of DN performed specifically by GPs should be addressed. Firstly, continuity of care, which is a fundamental aspect of primary care can augment the success of the procedure [39]. In our study, two GPs, a specialist and a resident, performed DN on their patients. Both had 3–4 years of experience with DN, but the specialist had worked in the same clinics for 8 years while the resident only worked in the clinic for 1 year. When DN was performed by the specialist, better results were observed with higher rates of immediate and continuous success. Although a meta-analysis indicated that experience of acupuncturist does not modify outcomes of DN [40], this might not be true to GPs. The impact of DN performed by a familiar physician might add to the success of the treatment, giving GPs a leverage on other health care providers providing this procedure. Secondly, access to care is essential to management of acute pain, which is another advantage of GPs over consultant physicians.

Preferences of patients might also be different when GPs perform DN compared to other health care workers; our study demonstrated that insertion of 1 needle (vs. multiple needles) was associated with short-term and continuous success, contrary to a meta-analysis that found the effect of acupuncture increased when more needles were inserted [40]. This may represent patients’ preferences for a shorter and more precise intervention during a GP visit compared to a physiotherapist consultation or a consultant physician, like an orthopedic specialist.

### Strengths

This study examines the effect of DN performed by GPs in their clinics. This is unlike most studies, which examine DN use by physiotherapists or physicians that are not necessarily GPs. As this procedure is widely and increasingly used by GPs, this is an important aspect of the research on the effectiveness of DN. The use of a validated scale for pain, the SF-MPQ, increases the validity of this study. Measuring the effect of DN both in short- and medium-term is another strength of this study.

### Limitations of the data

This study did not include a control group, and thus the treatment cannot be compared to other treatment options or to sham-therapy. Additionally, patients were treated by two GPs in the southern district of Israel; the population of patients does not represent the whole Israeli population, which may affect the results found. Both physicians who performed the DN also treat patients for other medical problems. This may influence patients’ satisfaction and pain. The specialty status of the GPs (one was a specialist and the other a resident) might also have impacted the results. Another limitation is the lack of follow-up with patients who declined to participate; thus, a possible selection bias might exist.

## Conclusion

DN is a good therapeutic option for GPs to aid patients suffering from acute pain. All SF-MPQ parameters of pain improved significantly, both in short- and medium-term. The physician who performed the procedure was associated with short-term and continuous success rates. Initial high intensity pain (VAS > 5) was associated with medium-term success. Considering it is a low-cost method and relatively easy skill to impart, it should be encouraged by policy makers to increase its availability to patients. This can be done by exposing students, residents and specialists to this skill. Further research should explore the effectiveness of DN shortly after having learned the method and by a larger group of GP participants in different settings. Future studies may prefer to choose algometry as a method to assess pain rather than questionnaires as we had chosen.

## Supplementary Information

**Additional file 1.** Dataset - IMS. 

## Data Availability

All data generated and analyzed during this study are included in this published article and its supplementary information files.

## Abbreviations

DN: Dry needling
GPs: General practitioners
IMS: Intra-muscular stimulation
MPS: Myofascial pain syndrome
MTrPs: Myofascial trigger points
SES: Socioeconomic status
SF-MPQ: Short-Form McGill Pain Questionnaire
VAS: Visual analog scale
